# Advances in understanding hypopituitarism

**DOI:** 10.12688/f1000research.9436.1

**Published:** 2017-02-22

**Authors:** Mareike R. Stieg, Ulrich Renner, Günter K. Stalla, Anna Kopczak

**Affiliations:** 1 Max Planck Institute of Psychiatry, Clinical Neuroendocrinology, Kraepelinstr. 2-10, D-80804 Munich, Germany

**Keywords:** hypopituitarism, combined pituitary hormone deficiency, isolated growth hormone deficiency, post-traumatic hypopituitarism, vascularization, Sheehan`s syndrome

## Abstract

The understanding of hypopituitarism has increased over the last three years. This review provides an overview of the most important recent findings. Most of the recent research in hypopituitarism has focused on genetics. New diagnostic techniques like next-generation sequencing have led to the description of different genetic mutations causative for congenital dysfunction of the pituitary gland while new molecular mechanisms underlying pituitary ontogenesis have also been described. Furthermore, hypopituitarism may occur because of an impairment of the distinctive vascularization of the pituitary gland, especially by disruption of the long vessel connection between the hypothalamus and the pituitary. Controversial findings have been published on post-traumatic hypopituitarism. Moreover, autoimmunity has been discussed in recent years as a possible reason for hypopituitarism. With the use of new drugs such as ipilimumab, hypopituitarism as a side effect of pharmaceuticals has come into focus. Besides new findings on the pathomechanism of hypopituitarism, there are new diagnostic tools in development, such as new growth hormone stimulants that are currently being tested in clinical trials. Moreover, cortisol measurement in scalp hair is a promising tool for monitoring cortisol levels over time.

## Introduction

Hypopituitarism is defined as a diminished function of the pituitary gland. First described in 1914 by Simmonds, it is also known as Simmonds’ disease ^1^. There are two main reasons for the hypofunction of the pituitary gland: it can result from pituitary dysfunction *per se* or from hypothalamic damage. In both cases, the production of pituitary hormones is diminished. When a single pituitary hormone is affected, this is called isolated pituitary deficiency. When two or more pituitary hormones are affected, this is referred to as multiple pituitary hormone deficiency. Panhypopituitarism is a state of reduction of all pituitary hormones.

Hypopituitarism may result from different causes and can be congenital or acquired. The most common causes are listed in Table 1. Depending on the specific anatomic lesion, patients present with different endocrine abnormalities. These either can be detected during newborn screenings such as congenital hypothyroidism or may present later in life (for example, with growth retardation or absence of puberty). As hypopituitarism may be associated with malformations of the central nervous system, such as malformation of the optic nerve, patients may present with different grades of sensory impairments. Acquired hypopituitarism can occur in all age groups and needs careful consideration of treatment indication depending on the nature and degree of the affected hormone axis.

**Table 1.** Most common causes of hypopituitarism.

Etiology	Underlying cause/disease (examples)
Congenital	• Idiopathic (without anatomical lesion or association with syndromic disease) • With anatomic lesion in the sella region (for example, primary empty sella syndrome or Rathke’s cyst) • Associated with malformations of the central nervous system (for example, septo-optic- dysplasia, Kallmann syndrome, and pituitary stalk interruption syndrome)
Acquired	• Pituitary tumor (mainly displacing macroadenoma) • Craniopharyngeoma • Transsphenoidal or transcranial surgery in the hypothalamo-pituitary region • Cranial radiation • Systemic cancer treatment • Traumatic brain injury • Sheehan’s syndrome • Apoplexy • Subarachnoid hemorrhage • Meningitis • Hypophysitis • Meningioma in the sella region • Lymphoma • Wegener’s granulomatosis • Hemochromatosis

## Genetic causes of congenital hypopituitarism

Despite recent advances, genetic causes are still not identified in the majority of patients with congenital hypopituitarism. Affected patients can be subdivided into two groups: one with concomitant association with extra-pituitary abnormalities (syndromic diseases) and the other presenting with hypopituitarism alone. Congenital hypopituitarism may be isolated with a loss of function of a single pituitary axis or presenting with dysfunction of two or more pituitary axes. However, initial diagnosis of isolated growth hormone deficiency (GHD) may frequently develop into multiple pituitary hormone deficiencies as described in a long-term observational study with a mean follow-up of more than 15 years ^2^. Analysis of the KIMS database (Pfizer International Metabolic Database) has shown that hypopituitarism in adults seems to be a dynamic condition where new hormone deficiencies may occur even years after initial diagnosis of isolated pituitary hormone deficiency ^3^.

The diagnosis of congenital causes for hypopituitarism can be challenging. Sequencing technologies may provide the underlying genetic cause. Sanger sequencing can be used if a single gene mutation is suspected because of the patient’s phenotype. More often, a multi-panel test seems to be more appropriate if no obvious clinical phenotype or magnetic resonance imaging finding is present. More recently, developed techniques such as next-generation sequencing and whole exome sequencing allow for the analysis of several relevant genes in parallel or coding regions of all genes, respectively. It has to be kept in mind that only 10% of cases with hypopituitarism can be explained by genetic alterations known so far ^4^. Epigenetic alterations, mosaicism, and oligonucleotide repeat expansions as well as alterations outside the coding regions cannot be detected by the above-mentioned sequencing techniques. However, owing to these improved diagnostic tools, there have been several causative genetic alterations identified in the last few decades, ranging from chromosomal abnormalities to single gene mutations ^4, 5^. The most recent publications will be summarized here briefly.

In general, alterations in transcription factors implicated in pituitary organogenesis can be pituitary-specific, such as mutations in *POU1F1*, or can affect different tissues, as is the case in *OTX2* mutations where pituitary deficiency is usually associated with severe eye abnormalities. In a recently published case report, an *OTX2* mutation was identified in a neonate presenting with adrenocorticotropic hormone deficiency, bilateral microphthalmia, and agenesis of the left internal carotid artery, expanding the spectrum of pathophysiology associated with *OTX2* mutations ^6^.

In patients with hypogonadotropic hypogonadism (in combination with anosmia, known as Kallmann syndrome), plenty of genetic alterations affecting appropriate GnRH action have been described. Mutation in the genes *FEZF1* ^7^, *SOX10* ^8^, and *FGF17*, *IL17RD*, *SPRY4*, *DUSP6*, and *FLRT3* ^9^ have been identified. Whether these single mutations alone are sufficient to cause disease remains unclear. For clinicians, a panel-testing approach in patients with hypogonadotropic hypogonadism/Kallmann syndrome is recommended ^5^.

In patients with isolated GHD, genetic alterations hindering the release of functioning growth hormone have been identified. Mutations in the pituitary-specific transcription factor *POU1F1* usually lead to an altered somatotropic, lactotropic, and thyreotropic axis. Recently, a heterozygous missense mutation in *POU1F1* was identified as a causal trigger in a family with isolated GHD ^10^. Moreover, a homozygous point mutation in the promotor region of the human growth hormone gene ( *GH1*) was firstly described as causative for isolated GHD in siblings with normal pituitary imaging ^11^. These two studies demonstrate the possibility of familial heredity that is either autosomal dominant or autosomal recessive.

The Italian Study Group on Genetics of multiple pituitary hormone deficiencies recently published a systematic review investigating the mutation frequency in the most common altered genes, *PROP1*, *POU1F1*, *HESX1*, *LHX3*, and *LHX4* ^12^. The calculated prevalence of defects in these genes was quite low in the Italian study cohort (2.9% in sporadic and 12.5% in familial cases), and a worldwide prevalence of 12.4% has been estimated (from 11.2% in sporadic up to 63% in familial cases) identifying *PROP1* as the most frequent mutated gene. Genetic alterations of *PROP1* are usually associated with non-syndromic pituitary deficiencies. Nevertheless, dysfunction of this transcription factor can cause a variety of different phenotypes probably due to residual activity of altered protein synthesis ^13^. Mutations in the non-pituitary-specific transcription factor *HESX1* are commonly associated with syndromic clinical features and have been linked to septo-optic dysplasia. However, dysfunction of *HESX1* may also present with a wide range of phenotypes ^14^, making targeted genetic diagnostics in patients with multiple pituitary hormone deficiencies even more challenging.

It must also be kept in mind that the detection of a mutant gene does not mean dysfunction of the encoded product *per se*. High-performing genetic sequence analysis enables the detection of a variety of genetic mutations and variants of unknown significance, as demonstrated in an analysis of the transcription factor *GLI2* ^15^. *GLI2* is a large polymorphic gene, and a relatively high frequency of *GLI2* mutations and variants were identified in patients with congenital GHD without further brain abnormalities. In mice, a link between *GLI2* mutations and prenatal ethanol exposure has been demonstrated, thereby implicating environmental factors in the etiology of *GLI2*-associated hypopituitarism ^15^. Furthermore, variability in penetrance and not-yet-identified gene–gene interactions underline the need for functional studies in patients with hypopituitarism.

In patients with congenital holoprosencephaly, a midline defect with variable phenotypes, several genetic alterations in the sonic hedgehog signaling pathway have been identified. Pituitary stalk interruption syndrome and isolated pituitary hypoplasia have recently been proposed as mild phenotypic variants of holoprosencephaly, as several genetic mutations in holoprosencephaly-associated genes have been proven in both entities. Affected genes comprise *TGIF1* and *SHH*, both relevant in sonic hedgehog signaling ^16^. In familial clustering of the disease, a homozygous mutation of *GPR161*, a gene coding for a G-protein-coupled receptor, was recently identified ^17^. Moreover, detection of a novel mutation in the *CDON* gene relevant in sonic hedgehog signaling ^18^ reveals the broad spectrum of implicating variants in pituitary dysgenesis and dysfunction.

## Hypopituitarism due to impaired hypophyseal blood supply

The anterior pituitary is one of the best-vascularized mammalian organs. Its high vessel density persists throughout the lifespan as the proper function of the pituitary is dependent on the rapid transportation of hypothalamic or peripheral factors from the bloodstream to the pituitary cells and the fast release of hypophyseal hormones into the blood ^19, 20^. The intrapituitary vessel density is reduced in *Prop1*-deficient mice, a model of pituitary hormone deficiency. However, as in these mice the development of hormone-producing cells is also impaired, it is not clear whether disturbed hormone transportation due to the reduced vessel density contributes to hypopituitarism in these mice ^21^.

In general, blood supply-dependent pituitary insufficiencies are rare ^22^. Mechanical ruptures of the long portal blood vessels, which connect the pituitary with the hypothalamus, have been suggested to contribute to pituitary insufficiency in patients with traumatic brain injury (see the next section). In patients with congenital pituitary stalk interruption syndrome, it is still not known to which extent an impaired portal blood vessel system contributes to the hypopituitarism seen in these patients ^23, 24^. Pituitary insufficiencies due to impaired hypophyseal blood supply are in most cases caused by compression of the long portal blood vessels or by pituitary apoplexy. Very often, these events are associated with or caused by pituitary tumors or local hemorrhages ^22, 25, 26^. Depending on the degree of blood flow impairment, a partial pituitary insufficiency can be observed in affected patients. This partial insufficiency can be reversible after normalizing the hypophyseal blood flow (for example, by removing the compressing structure) ^25, 27^. Severe apoplexy leading to intrapituitary hypoxia and necrosis of hormone-producing cells is a life-threatening condition and needs rapid and proper treatment ^22, 26^.

Reduced blood supply of the pituitary gland is attributed to Sheehan’s syndrome, a common cause of hypopituitarism in post-partum women. The enlargement of the pituitary gland during pregnancy in combination with massive post-partum hemorrhage leads to a significant reduction of arterial blood flow and causes pituitary necrosis ^28^. The prevalence of Sheehan’s syndrome might have decreased in recent years in some European countries and this is probably due to better obstetric care ^28^. In all other parts of the world, including the former Eastern European countries, the Middle East, the Mediterranean, and Africa, Sheehan’s syndrome is still one of the most common causes of hypopituitarism. Signs and symptoms are often unspecific, and this may cause a delay in or impair correct diagnosis. A diagnostic delay of about 20 years was described and recently confirmed in Turkey ^29, 30^. Recently, an increased expression of the coagulation factor V gene was reported in patients with Sheehan’s syndrome ^31^. However, gene polymorphisms in coagulation factors II and V and in tumor necrosis factor-alpha seemed not to be attributed to the pathogenesis of Sheehan’s syndrome ^32^. In general, the role of coagulation factors in Sheehan’s syndrome has to be further elucidated.

## Acquired hypopituitarism due to brain trauma

Acquired hypopituitarism can be observed in several neurological diseases. It was first described in a patient after traumatic brain injury leading to post-traumatic hypopituitarism ^33^. There are two distinct pathomechanisms causing post-traumatic hypopituitarism ^34^. The pituitary gland can be affected directly at the moment of the trauma (for example, by blast injuries, skull fractures with involvement of the sella turcica, or pituitary stalk disruptions). Not only do direct damages of the pituitary gland lead to hypopituitarism but also secondary effects of the trauma such as compression of the pituitary gland by brain swelling, impairment of the vascularization through swelling or vasospasms, and consecutive cell death of pituitary cells result in post-traumatic hypopituitarism.

Post-traumatic hypopituitarism may have causes other than just a single event. Repetitive concussions in contact sports such as football ^35^ or boxing ^36^ may also result in post-traumatic hypopituitarism.

As described above, the perfusion of the pituitary gland is crucial for the survival of pituitary cells. From an anatomical perspective, acidophil growth hormone-producing cells are located mainly at the periphery of the pituitary gland, thus making them sensitive to perfusion deficits and hypoxia. This fact contributes to the finding that somatotropic insufficiency is the most frequent hormonal deficit after brain injury ^37– 39^, especially in chronic patients ^40, 41^.

Recently, this finding has been controversially discussed. In line with recent guidelines, GHD has to be defined by stimulation tests such as the insulin tolerance test or the growth hormone-releasing hormone (GHRH)–arginine test ^42^. In a Danish National Study, only a low incidence of GHD of less than 1% after brain injury was reported when patients received a confirmatory stimulation test ^43^. Hence, the high prevalence and especially the relevance of post-traumatic GHD have been questioned ^44^.

This controversial discussion has extended to patients with subarachnoid hemorrhage (SAH). The first reports showed high prevalence rates of hypopituitarism after SAH of 47.5% ^38, 45^, especially of GHD with a prevalence of 20 to 25% in chronic patients ^46^. Recent findings suggest that these high prevalence rates might be overestimated ^47, 48^. The inclusion of different patient cohorts with distinct characteristics such as different severity grade of TBI/SAH and the use of different diagnostic tests might contribute to these controversial findings ^49^. This outlines that test stringency, especially for GHD, is very important in these patient cohorts. Additional diagnostic tools would be helpful. Recently, circulating microRNAs were identified in patients with post-traumatic hypopituitarism, suggesting that the microRNAs miR-126-3p and miR-3610 may act as potential biomarkers for hypopituitarism after TBI ^50^.

The diagnosis of hypopituitarism may be difficult in patients after brain injury, since signs and symptoms of pituitary dysfunction may overlap with chronic sequelae of TBI and SAH. However, it was shown that pituitary dysfunction had no effect on the commonly reported fatigue following SAH ^51^. Moreover, endocrine disturbances such as hypogonadism and hypothyroidism may occur as physiological stress responses to critical illness in patients after very severe brain injury, even long after the trauma ^52^. Hence, pathological laboratory findings do not automatically implicate the presence of post-traumatic hypopituitarism ^53^.

But not only TBI and SAH may lead to hypopituitarism. Pituitary dysfunction also occurs after stroke with an estimated prevalence rate of about 19%. Thus, hypopituitarism seems to be less frequent in stroke patients than in patients after TBI or SAH ^54^. Despite the impact of stroke on morbidity and mortality, research on hypopituitarism after stroke has been very limited in recent years ^55^. Moreover, hypopituitarism may rarely occur after infectious diseases like encephalitis or meningitis ^56, 57^ and should be considered in these patients presenting with an unusual course.

In addition, cranial radiation is the most common cause of iatrogenic hypopituitarism ^58^. The radiation of brain or neck tumors may affect the pituitary gland, thus leading to hypopituitarism ^59^. In children with acute lymphoblastic leukemia, GHD seems to be the most common hormonal deficit after cranial radiation ^60^. In adults, an increasing prevalence of hypopituitarism from 12.5% after 2 years up to 90% 15 years after radiation has been reported ^61^. As hypopituitarism due to cranial irradiation will increase as a consequence of more cancer patients receiving this treatment, establishing well-functioning collaboration between endocrinologists and oncologists is essential for adequate follow-up for these patients.

The role of autoantibodies in the pathophysiology of secondary hypopituitarism has been discussed in more detail in recent years. Damage of the pituitary integrity might expose pituitary antigens, leading to autoimmune-mediated damage of the pituitary gland in the presence of pituitary antibodies. However, the impact of pituitary antibodies on the pathophysiology of secondary hypopituitarism after brain injury remains unclear. There are several findings indicating that secondary hypopituitarism is associated with the presence of autoantibodies. These findings seem to be stable even years after the event ^62^. However, it has to be kept in mind that there is no standardization or even proper validation of autoantibody assessment and such findings have not been confirmed by other research groups so far ^63^.

## Auto-antibodies of the pituitary gland and the hypothalamus

Autoimmunity has recently been discussed as a further pathomechanism of hypopituitarism. Two different types of antibodies play a role in hypopituitarism: antipituitary antibodies and antihypothalamus antibodies.

Although antipituitary antibodies were first described in 1965 in a patient with Sheehan’s syndrome ^64^, it is still not clear what their exact target is ^63^. Alpha-enolase, gamma-enolase, the pituitary gland-specific factors 1 and 2, and corticotroph-specific transcription factor (TPIT) have been discussed as possible targets of antipituitary antibodies ^65^. The measurement of antipituitary antibodies is complicated by different methodological problems, and the clinical use of antipituitary antibodies and antihypothalamus antibodies is very limited so far because of the lack of standardized and validated methods. In autoimmune hypophysitis, also known as lymphocytic hypophysitis, the measurement of antipituitary antibodies can be considered, but sensitivity and specificity are low ^65^.

Ricciuti *et al*. recently showed that antipituitary antibodies are more common in patients with pituitary diseases (24%) when compared with healthy controls (5%), but a discrimination of pituitary diseases has not been possible ^66^.

De Bellis *et al*., in a longitudinal study, recently demonstrated that children with isolated GHD and high antipituitary antibody titers were not likely to show remission ^67^. Furthermore, antipituitary antibody-positive patients were at risk for delayed puberty due to hypogonadotropic hypogonadism. The presence of antipituitary antibodies may precede hypogonadism ^68^.

Less is known about antihypothalamus antibodies. These antibodies seem to target corticotropin-releasing hormone (CRH)-secreting cells in the hypothalamus ^69^. The presence of antihypothalamus antibodies was described in amateur boxers with hypopituitarism ^36^ as well as in patients with idiopathic hypopituitarism ^70^. In general, the literature on antihypothalamus antibodies is very scarce. A standardization and proper validation of methods are necessary for a better understanding of these results. This would also enable wider use of these autoantibody assessments.

## Drug-induced hypopituitarism

The discussion of iatrogenic hypopituitarism has focused mainly on induction of autoimmune hypophysitis by immune checkpoint-inhibiting medication used in cancer treatment. A recently published review provides a good overview of the prevalence and pathophysiology of ipilimumab-induced autoimmune hypophysitis associated with pituitary dysfunction ^71^. In brief, the incidence of hypophysitis is less than 10% with endocrine dysfunction occurring approximately 9 weeks after the initiation of therapy on average. Drug cessation and high-dose glucocorticoid administration have been proposed to manage these side effects. Continuation of the therapy may lead to hypopituitarism and can necessitate pituitary hormone substitution.

## Recent developments in the diagnosis of hypopituitarism

In the case of suspected hypopituitarism, testing for the function of a single pituitary axis should be performed stepwise and according to the clinical presentation. Guidelines (for example, clinical practice guidelines of the Endocrine Society ^72^) provide advice for a reasonable screening and use of appropriate tests. However, there are some new developments in the diagnosis of isolated pituitary deficiencies, which will be highlighted here.

Analysis of human scalp hair has gained more attention in recent years. Originally used for consumption behavior in a forensic context, analysis of the content of steroids, mainly cortisol, has been proposed as a promising biomarker for individual cortisol exposure over a defined period of time ^73^. The sampling procedure is easy and non-invasive, and samples can be stored at room temperature for years. Determination of cortisol content in human scalp hair was recently used for retrospective determination of disease onset in a patient with primary adrenal insufficiency ^74^. Furthermore, measurement of hair cortisol content in patients with corticotroph insufficiency could be considered as a useful tool for therapy monitoring rather than as a diagnostic tool ^73^.

The diagnosis of GHD requires confirmation using a growth hormone stimulation test. The insulin hypoglycemia test represents the gold standard, but there are several contraindications and the test procedure is physically demanding for patients. Body mass index-related cutoff values are used in the GHRH–arginine test ^75^, and recent studies have emphasized new cutoff levels for the glucagon stimulation test, which seems to be an acceptable alternative to the insulin hypoglycemia test ^76^. Furthermore, new growth hormone stimulants for oral application are currently being validated in clinical trials ^77, 78^.

## Conclusion and future perspective

In patients with acquired pituitary insufficiency, understanding of the pathomechanisms has increased in recent years. The importance of a proper blood supply and the role of autoimmunity have been discussed. A more profound understanding of the underlying causes leading to secondary hypopituitarism may lead to recommendations for rational screening of hypopituitarism in more precisely defined risk populations and therefore may prevent under- or over-diagnosis.

Gene sequencing methods have markedly improved. New diagnostic tools led to the discovery of different genetic alterations in patients with congenital hypopituitarism. These mutations mainly affect genes important for pituitary ontogenesis. However, the diagnosis of congenital causes for hypopituitarism can be challenging and remains unsolved in the majority of patients. Depending on the phenotype and medical history of a single patient, an individual approach for stepwise genetic testing is essential. Increasing our understanding of molecular pathways relevant in pituitary function also includes expanding on current knowledge of pituitary cell differentiation and proliferation. Very recently, it was shown that pituitary cells seem to have the capability to regenerate, which provides a promising approach for therapeutic strategies for patients with permanent hypopituitarism ^79^. Nevertheless, activation of pituitary stem cells and cell transplantation in the treatment of pituitary insufficiency is still a long way off ^80, 81^.
